# Case of Aneurism of the Aorta, with the Physical Signs

**Published:** 1853-01

**Authors:** Austin W. Nichols

**Affiliations:** Med. Student


*Case of Aneurism of the Aorta, with the Physical Signs.* Reported in a letter from Austin W. Nichols, Med. Student.

*Sept. 4th*, 1852. Before the surgical clinic of a medical college in this city, there appeared a man, aged about 45, his neck enormously distended, and his breathing labored. He stated, that for several weeks he had been engaged in digging cellars, his feet being constantly in the wet. This swelling came on gradually, without any previous sickness. The swelling pitted on pressure and was due to serous effusion into the areolar tissue of the neck. He had no fever, there was no redness; and the only symptom was the quickened and forced respiration. The distinguished surgeon pronounced it, a case of œdematous erysipelas. He directed to be applied locally, a saturated solution of nitrate of silver; and internally, he gave diuretics as digitalis, squill, and calomel; and as a drink, he prescribed juniper-berry tea. On the 11th, he appeared at the same clinic much better, and was directed to continue the internal medicines. I did not see the patient again till my visit at the Pennsylvania Hospital, on the 10th of November. I was then surprised to see the man with his neck more distended and his breathing quicker and more laborious than ever. Dr. Wood, the attending physician, uncovered the chest and arms of the patient; the superficial veins were much enlarged and could be seen distinctly; but the veins of the abdomen and lower extremities were healthy and not at all enlarged. There was no ascites nor anasarca. On percussion, flatness was found between the second and fourth ribs to the right and left and over the sternum. The left side was flat as far down as the fifth rib; and the right side was flat as far as the nipple. On auscultation, the sounds of the bronchi and lungs were found to be healthy or natural, though somewhat obscured by the following murmurs. On listening to the cardiac sounds, the first was replaced by a long continuous murmur occupying the time of the first natural sound and part of the time allotted to the second natural sound; the second sound was replaced by another continuous murmur occupying the time of the second natural sound and part of the time of the interval between the sounds. The first murmur was heard most distinctly over the mitral valves; the second, over the aortic or semilunar valves. These sounds were due to an imperfect closure of the valves on the left side of the heart. The sounds on the right side, though obscured, were not very unnatural. Particular attention, however, was not given to the state of this side of the organ. No aneurismal thrill was present in the course of the aorta or its large branches. I may here add, that when the patient stooped forward, his face became dark, his head swelled, and giddiness came on. On feeling the pulse of the right arm, it was found to be much feebler than that of the left arm. The diagnosis was easily arrived at; there was disease of the valves of the left side of the heart, and there was an aneurism of the aorta or of its large branches. This aneurismal tumor pressed upon the superior cava and impeded the return of the blood from the upper extremities. The treatment was but palliative; diuretics, as digitalis, squill, bitartrate of potassa, being given. On the 20th of November, I saw the pathological specimens from the above case; death had taken place slowly, not from rupture of the aneurism. The mitral and semilunar valves of the left side of the heart, were much thickened by a deposit semi-cartilaginous: the ventricle was dilated and thickened to a very great extent. The greater part of the ascending aorta and the entire arch, was an aneurismal sac; this was a mere dilatation of all the aortic coats, and not a dilatation of the outer one alone with rupture of the inner coats; the branches of the aorta were given off from this enlarged aorta as usual. The sac would hold the two fists with ease; its inner surface was covered with an atheromatous deposition; on its external surface, the face toward the sternum had thrown out coagulable lymph and caused an adhesion to the sternal bone. The valves on the right side of the heart, were slightly thickened; the cavities were of the natural size. But the superior vena cava, for near its whole length, was greatly thickened; its walls had been pressed upon and had adhered; the channel of the vein was reduced to about one line in diameter. Thus was the diagnosis of this singular case verified.

*Jefferson Med. College*, Nov. 22, 1852.

